# Protein-Specific Features Associated with Variability in Human Antibody Responses to *Plasmodium falciparum* Malaria Antigens

**DOI:** 10.4269/ajtmh.17-0437

**Published:** 2017-10-23

**Authors:** Eugene W. Liu, Jeff Skinner, Tuan M. Tran, Krishan Kumar, David L. Narum, Aarti Jain, Aissata Ongoiba, Boubacar Traoré, Philip L. Felgner, Peter D. Crompton

**Affiliations:** 1 Laboratory of Immunogenetics, National Institute of Allergy and Infectious Diseases, National Institutes of Health, Rockville, Maryland;; 2 Division of Infectious Diseases, Department of Medicine, Indiana University School of Medicine, Indianapolis, Indiana;; 3 Laboratory of Malaria Immunology and Vaccinology, National Institute of Allergy and Infectious Diseases, National Institutes of Health, Bethesda, Maryland;; 4 University of California, Irvine, Irvine, California;; 5 Mali International Center of Excellence in Research, University of Sciences,Techniques and Technologies of Bamako, Bamako, Mali

## Abstract

The magnitude of antibody responses varies across the individual proteins that constitute any given microorganism, both in the context of natural infection and vaccination with attenuated or inactivated pathogens. The protein-specific factors underlying this variability are poorly understood. In 267 individuals exposed to intense seasonal malaria, we examined the relationship between immunoglobulin G (IgG) responses to 861 *Plasmodium falciparum* proteins and specific features of these proteins, including their subcellular location, relative abundance, degree of polymorphism, and whether they are predicted to have human orthologs. We found that IgG reactivity was significantly higher to extracellular and plasma membrane proteins and also correlated positively with both protein abundance and degree of protein polymorphism. Conversely, IgG reactivity was significantly lower to proteins predicted to have human orthologs. These findings provide insight into protein-specific factors that are associated with variability in the magnitude of antibody responses to natural *P. falciparum* infection—data that could inform vaccine strategies to optimize antibody-mediated immunity as well as the selection of antigens for sero-diagnostic purposes.

## INTRODUCTION

In the context of natural infections as well as vaccination with attenuated or inactivated microorganisms, the magnitude of antibody responses varies across the individual antigens that constitute the microorganism. It is unclear whether the heterogeneity in antibody responses is associated with specific features of proteins, such as subcellular location, relative abundance, molecular weight (MW), degree of polymorphism, or whether a protein is predicted to have human orthologs. In the case of antibody responses to malaria, until recently, technical limitations, such as low-throughput serological assays and traditional cloning and protein expression methods that made < 0.5% of *Plasmodium falciparum* proteins available for analysis^1^ precluded systematic, unbiased analyses of the relationship between protein-specific features and host antibody responses. Indeed, most prior efforts to understand the mechanisms underlying immunodominance have focused on differences in immune responses between epitopes within a given antigen rather than differences between antigens of a given microorganism.^2^ For example, prior studies have examined the link between immunodominance and antigen-B cell receptor binding affinity,^3^ epitope accessibility, hydrophilicity, and mobility,^4^ as well as variation in antigen processing and presentation to CD4^+^ T cells via peptide-MHC II complexes.^5–10^

While these features of B- and T-cell epitopes clearly influence immunodominance at the single antigen level, only recently has the genomics-based technology become available to examine protein-specific factors that underlie variability in antibody responses across the entire proteome of important pathogens. For example, a study in which serum samples from subjects with tuberculosis were probed against a protein microarray containing the full *Mycobacterium tuberculosis* proteome (4,099 proteins) showed enrichment of antibody responses directed against secreted *M. tuberculosis* proteins.^11^ Similarly, a study that probed serum samples from subjects with brucellosis against a protein microarray with 3,046 *Brucella melitensis* proteins showed enrichment of antibodies targeting secreted and membrane-associated proteins.^12^ Similar analyses have yet to be applied to eukaryotic pathogens, such as *Plasmodium* parasites, the causative agents of malaria. This is particularly relevant to *P. falciparum* malaria, as several vaccine strategies involve whole parasites that are either radiation attenuated,^13^ genetically attenuated,^14,15^ or given under chemoprophylaxis.^16^ Indeed, recent studies reveal heterogeneity in the magnitude of antibody responses to individual proteins that constitute these whole organism vaccine candidates.^13,16^ Nonetheless, there have been no published systematic attempts to understand the protein specific factors underlying the variability in antibody responses to *P. falciparum*. Here, we took advantage of *P. falciparum* protein microarray technology,^17^ publicly available protein annotation databases,^18,19^ and a cohort study conducted in an area of intense seasonal malaria to examine protein specific factors associated with antibody immunodominance in the context of natural *P. falciparum* infection.

## MATERIALS AND METHODS

### Ethical approval.

This study was approved by the Ethics Committee of the Faculty of Medicine, Pharmacy and Odonto-Stomatology, University of Bamako, Mali, and the Institutional Review Board at the National Institute of Allergy and Infectious Diseases, National Institutes of Health (NIAID protocol 11-I-N126). Plasma from anonymous healthy U.S. adult volunteers was obtained from the General Clinical Research Center at the University of California, Irvine (UCI IRB protocol 2007-5896). A written informed consent was obtained from adult participants and parents or legal guardians of participating children.

### Study participants.

**Figure 1.**
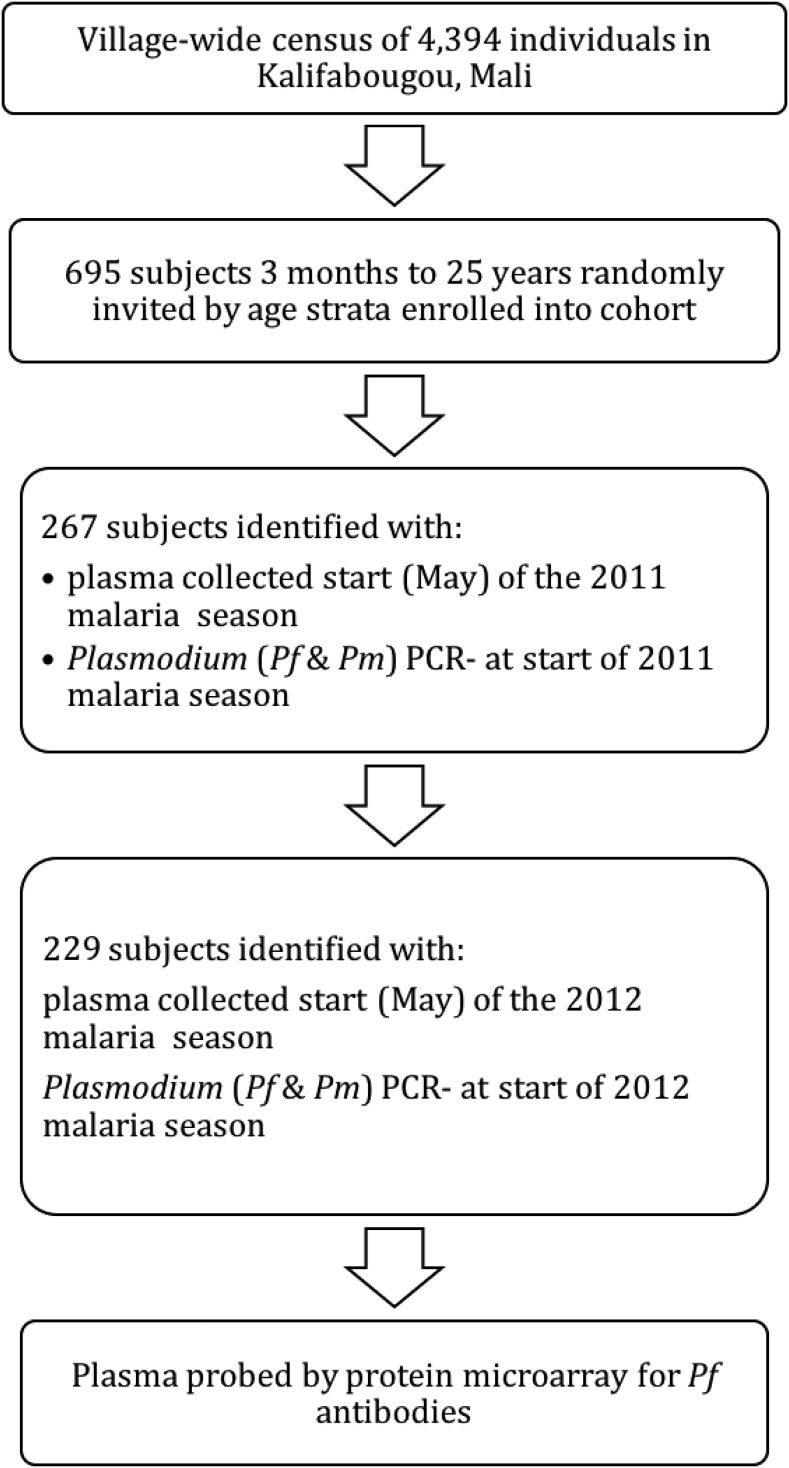
Study design flow chart. Sequence of selection events made to arrive at 234 study subjects.

This study was conducted in Kalifabougou, Mali, where intense *P. falciparum* transmission occurs from June through December each year, whereas transmission is negligible during the dry season from January through May. A detailed description of the study site and cohort design has been published elsewhere.^20^ At the end of the dry season in May 2011, we enrolled 695 healthy subjects aged 3 months to 25 years in this ongoing cohort (Figure 1). The disproportionate sample size of age groups reflects the design of this study that focuses on older children as they transition from malaria susceptibility to immunity. Exclusion criteria at enrollment included a hemoglobin level < 7 g/dL, axillary temperature ≥ 37.5°C, acute systemic illness, underlying chronic disease, use of antimalarial or immunosuppressive medications in the past 30 days, or pregnancy. For this analysis, we focused on an age-stratified subset of subjects from the cohort (*N* = 267) with ages ranging 3 months to 25 years of age whose fingerprick blood samples were negative for *Plasmodium* by polymerase chain reaction (PCR) at enrollment. Of these 267 subjects, 229 were also negative for *Plasmodium* by PCR after 1 year at the end of the dry season in May 2012. Paired plasma samples collected from subjects at these two timepoints (May 2011 and May 2012) were analyzed by protein microarray.

### Experimental design and statistical rationale.

A total of 515 experimental plasma samples (267 from May 2011, 234 from May 2012, and 14 replicates) were probed by protein microarray. In addition, 14 experimental replicates were also probed (2.8% of the experimental samples). To confirm reproducibility of the array, a total of 20 reference plasma replicates from Papua New Guinea were also probed (two replicates for every 96 samples). To minimize confounding from potential nuisance effects from array printing (such as a pad, slide, or batch printing effect) or assay performance technique during each run, the assay sequence of experimental samples and experimental replicates was randomized to evenly distribute samples by timepoint, age group, and sex across the sequence of protein microarray probing. To assess specificity of the microarray to *P. falciparum* infection, we included eleven negative control samples from malaria naive adults 20 to 59 years of age residing in Orange County, CA (with 11 probed in an earlier experiment with the same printed batch of microarrays, and one duplicate embedded within experimental samples). Power and sample size analysis was not used in this study as the samples are from a previously established protocol as described previously, and the maximum number of samples for the relevant age groups was used.

### Plasma samples.

Blood samples were drawn by venipuncture into tubes containing sodium citrate (Vacutainer CPT; BD Biosciences, San Jose, CA), stored in iceboxes below room temperature, and then transported to the laboratory within 12 hours. Plasma was separated by centrifugation and stored at −80°C.

### Detection of *P. falciparum* infections.

A two-step nested PCR was performed to amplify *Plasmodium* DNA directly from dried blood spots on filter paper. Detailed methods for the detection of *P. falciparum* by PCR are described elsewhere.^20^

### Antibody profiling by protein microarray.

The *Pf*1000 protein microarray (Antigen Discovery, Inc., Irvine, CA) containing 1,087 proteins representing ∼23% of the *P. falciparum* proteome was used to probe plasma samples. Proteins < 1,014 amino acids (*N* = 269) were expressed as full-length proteins, whereas proteins > 1,014 amino acids (*N* = 818) were expressed as polypeptide fragments. For simplicity, “microarray proteins” refers to both full-length proteins and polypeptides. The 1,087 microarray proteins were chosen from a larger previously constructed 4,528 protein array (*Pf4528*) representing 60% of the proteome in a downselection process. Sera from 20 *P. falciparum*-exposed adults each in Papua New Guinea, Kenya, and Mali, and 10 malaria-naive adults in the United States were probed with this microarray. Proteins were identified and placed into tiers by seroreactivity in: 1) all countries, 2) > 50% of countries, 3) < 50% of countries, and 4) proteins of significance from other analyses. Significant proteins were consecutively selected starting from the first tier until 1,087 proteins/polypeptides were identified.

The downselected proteins were expressed in a process^21^ that consists of PCR amplification of corresponding open reading frames of the 3D7 strain of *P. falciparu*m, in vivo recombination cloning into an *Escherichia coli* vector, in vitro transcription/translation, and printing of the protein product onto nitrocellulose-coated glass slides. Each slide was composed of eight separate microarrays (pads) against which one plasma sample was probed. Microarray probing is described in detail elsewhere,^17^ which in summary consisted of applying each 1:200 diluted individual plasma sample to one microarray, followed by a biotin-conjugated goat anti-human immunoglobulin G (IgG) secondary antibody and fluorescently labeled streptavidin conjugate. IgG reactivity was quantified by a microarray scanner as a unitless relative signal intensity.

### Measurement of femtomolar abundance of merozoite protein.

For investigation of the relationship between protein abundance and IgG reactivity, wild-type merozoites were obtained from the rupture of continuously cultured schizonts through cell-sieving and collection and prepared for liquid chromatography/mass spectrometry (LC/MS^E^) analysis described elsewhere.^22^ In brief, 4 × 10^8^ merozoites were suspended in 50 mM ammonium bicarbonate (pH 8.5) with 0.06% RapiGest™ (Waters Corp., Milford, MA). Disulfide bonds in merozoite protein were reduced with 10 mM dithiothreitol at 60°C for 30 minutes, followed by alkylation of free cysteine residues with 30 mM iodoacetamide at room temperature for 30 minutes, and then digested overnight with trypsin at 37°C. A two-dimensional reverse phase chromatograph with an online Synapt G2-S mass spectrometer (Waters Corp.) measured femtomolar abundance.

### Data analysis.

**Figure 2.**
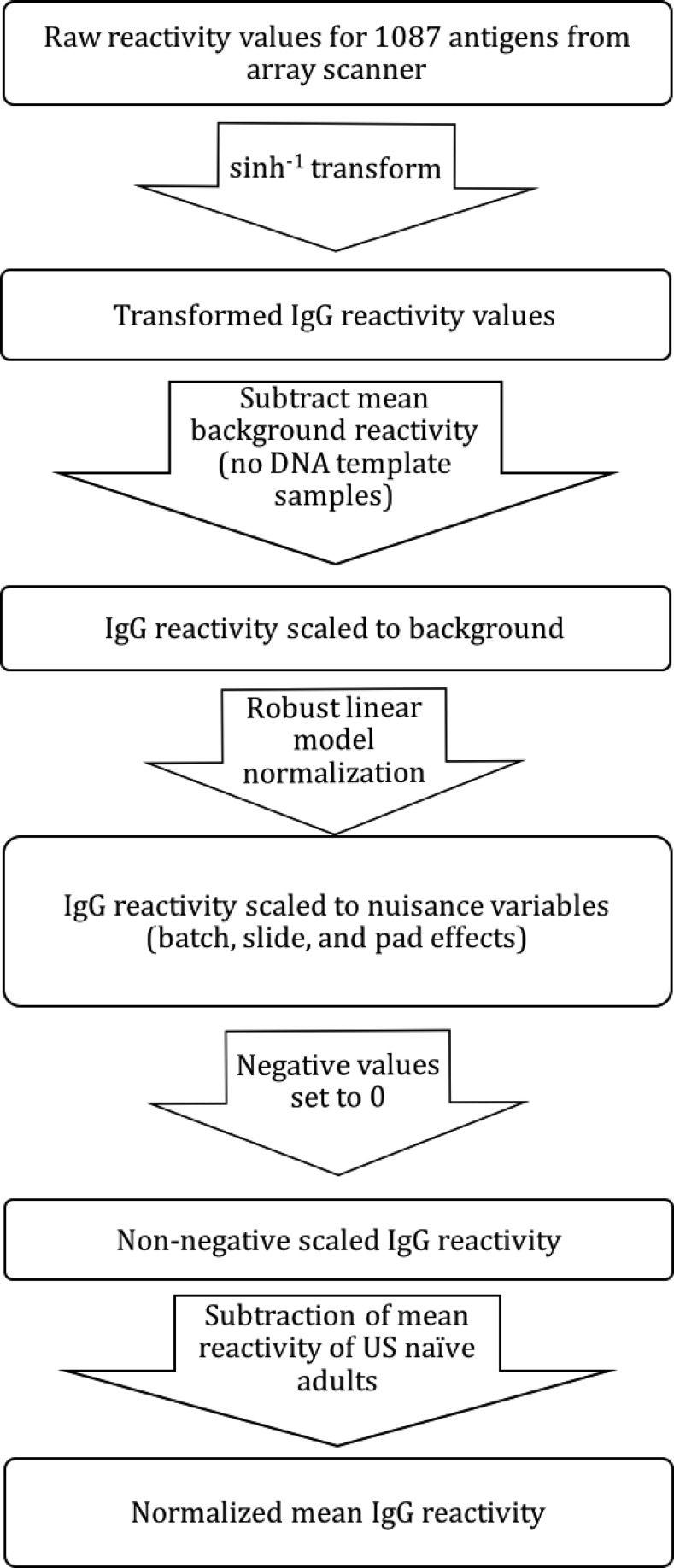
Data normalization flow chart. Sequence of steps made to normalize microarray reactivity to scale for background signal, nuisance variables, and adjustment for non-*Plasmodium falciparum* specific antibody binding.

R statistical software (version 3.2.3) was used to perform data analysis (Figure 2). Unless otherwise specified, IgG reactivity refers to normalized mean IgG reactivity in all study subjects in May of 2011. A sinh^−1^ transform (*f*(*x*) = sinh^−1^(*a* + *bx*), *a* = 1, *b* = 50) was used to transform measured IgG reactivity values for 1,087 antigens into a normalized distribution. To account for cross reactivity to background protein (i.e., protein byproducts of the expression system), mean NoDNA reactivity (represented by spots without DNA template in the plasmid vector) was subtracted from these transformed values, which were then scaled for nuisance variables specific to the protein microarray (batch, slide, and pad effects) using robust linear model normalization.^23^ Negative values (indicating reactivity below that of the negative control samples without DNA template) were set to zero. Reactivity of plasma from naive individuals to malaria protein would suggest the presence of non-*P. falciparum* specific antibodies. To adjust for potential nonmalaria specific antibodies found in both US naive and experimental plasma samples, mean nuisance scaled reactivity values of 11 US naive adults were subtracted from mean nuisance scaled values for the 267 subjects for each protein. We called this adjusted mean reactivity as normalized mean IgG reactivity, which was used as the response variable in subsequent analysis.

Data for each proteomic feature were obtained through publicly available annotations^18,19^ with the exception of merozoite protein abundance (ex vivo molar amounts), as described earlier. Microarray proteins were mapped to Gene Ontology Terms by cellular component using the Princeton Generic Gene Ontology Term Mapper^24,25^ referencing the GeneDB gene association file (April 1, 2015) for *P. falciparum* strain 3D7,^19^ then mapped to their respective highest level parent terms: intracellular, plasma membrane, and extracellular. The intracellular region was defined as any component within but not including the plasma membrane; the plasma membrane as the phospholipid bilayer and associated proteins separating the cell from its external environment; and the extracellular region as the space external to the parasite plasma membrane including the host cell environment outside an intracellular parasite. Presence of human orthologs was determined through the orthoMCL algorithm which clusters proteins into ortholog groups by BLAST similarity.^26^ Each proteomic feature was treated as a factor (if categorical) or covariate (if continuous) in a simple linear model with normalized mean reactivity for each protein as the dependent variable. Continuous proteomic features were also treated as factors by categorizing values in a mixture of densities model (R package *mixtools*^27^) with two normal distributions with a cutoff of two standard deviations above the mean of the lower normal distribution. Comparisons between factor levels were made using single-step adjusted contrasts from a linear model, and for continuous covariates, a simple regression line was estimated. Comparisons were also made for each factor by stratifying subjects into age group, from which normalized mean reactivity was compared using single-step adjusted contrasts from a linear mixed-effects statistical model (R package *lme4*^28^) with each subject as a random effect.

To identify collinearity, we performed Pearson χ^2^ testing for each possible pairing of proteomic features treated as factors. We then constructed multiple linear regression models using all significant proteomic features and interaction variables for factors previously determined not to be independent by Pearson χ^2^ testing using May 2011 data as a training set. Bidirectional stepwise regression was performed to determine relevant factors/covariates with nonrelevant factors/covariates removed. The final models were validated using May 2012 data.

## RESULTS

### IgG reactivity across individual *P. falciparum* proteins is markedly heterogeneous.

**Figure 3.**
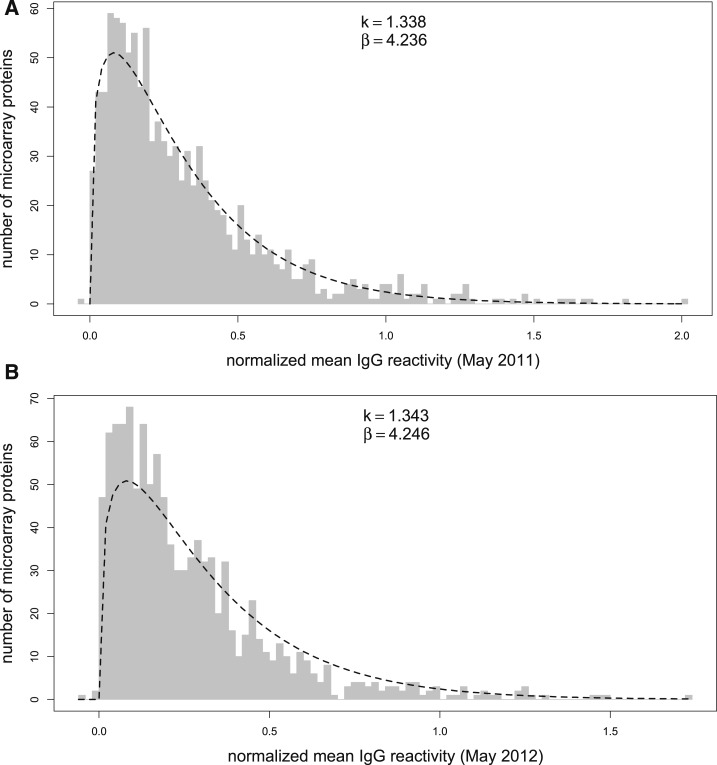
The level of Immunoglobulin G (IgG) reactivity to individual *Plasmodium falciparum* proteins is heterogeneous. (**A**) Histogram of normalized mean IgG reactivity to 1,087 *P. falciparum* microarray proteins from plasma collected in May 2011 from 267 subjects in Kalifabougou, Mali, in increments of 0.02. Positive normalized mean IgG reactivities fit a gamma distribution (*k* = 1.338, standard error = 0.052, β = 4.236, standard error = 0.1981) with predicted values shown by a dashed line. (**B**) Histogram for plasma collected in May 2012 from 229 subjects with fitting of positive normalized mean IgG reactivities to a gamma distribution (*k* = 1.343, standard error = 0.0521, β = 4.246, standard error = 0.1987).

Characteristics of 267 study subjects are shown in Table 1. Of these 267 subjects, 229 were also negative for *Plasmodium* by PCR 1 year later at the end of the dry season in May 2012. The antibody profiles of plasma samples collected from subjects at these two timepoints (May 2011 and May 2012) were analyzed by a protein microarray containing 1,087 *P. falciparum* proteins. This analysis revealed that IgG reactivity levels are markedly heterogeneous across individual *P. falciparum* proteins—ranging from nonspecific reactivity at the level of US naive samples (0) to two orders of magnitude above these negative controls (2.0). A similar pattern was observed in the same cohort 1 year later in May 2012 (Figure 3).

**Table 1:** Baseline characteristics of all 267 study participants by age group

	Age group						
Characteristic	< 2	2–4	5–7	8–10	11–17	≥ 18	All
Female sex	33	20	29	36	6	7	131
Hgb AC	3	5	4	13	1	1	27
Hgb AS	6	6	6	10	1	2	31
Hgb SC	0	0	0	1	0	0	1
Febrile malaria*	33	41	40	46	7	4	171
Total	67	54	51	74	11	11	229

* Febrile malaria defined as axillary temperature of 37.5°C, ≥ 2,500 asexual parasites/μL of blood, and no other cause of fever discernible by physical exam.

### IgG reactivity to extracellular and plasma membrane proteins is higher than IgG reactivity to intracellular proteins.

**Figure 4.**
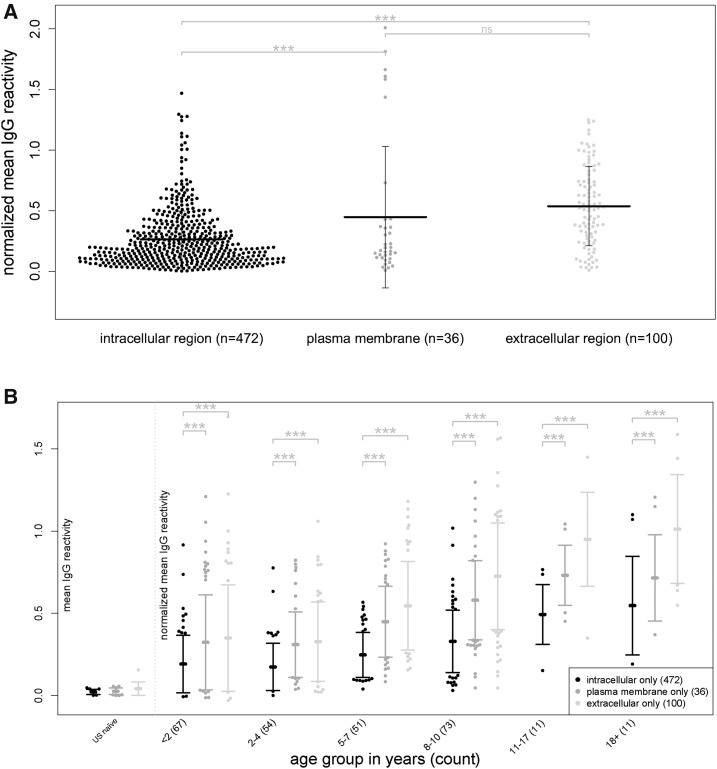
Immunoglobulin G (IgG) reactivity to extracellular and plasma membrane proteins is higher than IgG reactivity to intracellular proteins. (**A**) Mean IgG reactivities to individual *Plasmodium falciparum* proteins classified by Gene Ontology parent cellular component (intracellular, extracellular, and plasma membrane) in plasma samples of 267 subjects. Vertical bars represent standard deviation of mean of reactivities (bold horizontal line). (**B**) Mean IgG reactivities to individual *P. falciparum* proteins classified by Gene Ontology parent cellular component in plasma samples of 267 subjects stratified by age. All data are normalized to mean IgG reactivity of U.S. adults. Multiple single-step adjusted comparisons were made from a linear model in (**A**) and from a linear mixed-effects model in (**B**). ****P < 0.0001, ***P < 0.001, **P < 0.01, *P < 0.05, ns = not significant.

We first examined the relationship between IgG reactivity and the subcellular location of the proteins on the microarray. Of the 861 full-length proteins represented on the microarray, 735 mapped to generic cellular component Gene Ontology Terms (locations at the level of subcellular structures and macromolecular complexes found across all domains of life), whereas 608 mapped exclusively to their respective highest level parent terms: intracellular, plasma membrane, and extracellular. Of note, no proteins mapped exclusively to the parasitophorous vacuolar space or membrane. We found that IgG reactivity to extracellular and plasma membrane proteins was significantly higher than IgG reactivity to intracellular proteins (*P* < 0.001; Figure 4A), a finding that remained significant within each of the age strata (*P* < 0.001; Figure 4B).

### IgG reactivity correlates with protein abundance.

**Figure 5.**
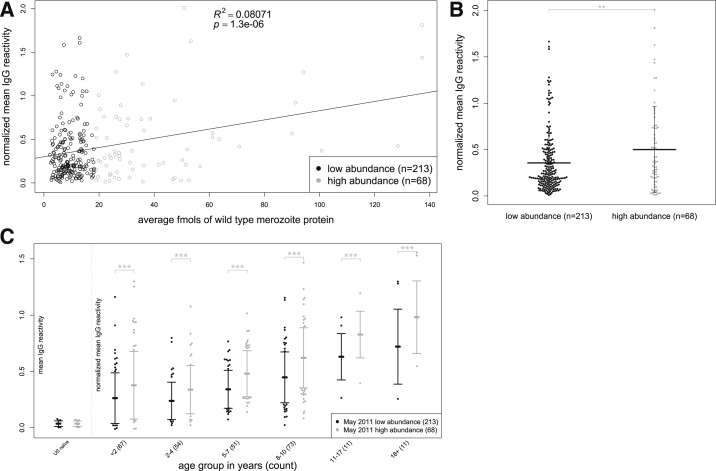
Abundance of merozoite proteins correlates with Immunoglobulin G (IgG) reactivity (**A**) Scatterplot showing mean IgG reactivity (in protein microarray data for 267 subjects) vs. mean abundance in femtomoles by mass spectrometry for 281 merozoite proteins (dots). (**B**) Mean IgG reactivities to low abundance (*N* = 213) vs. high abundance proteins (*N* = 68). Vertical bars represent standard deviation from mean. (**C**) Mean IgG reactivities to low abundance (*N* = 213) vs. high abundance proteins (*N* = 68) stratified by age. All data are normalized to mean IgG reactivity of U.S. adults. Scatterplot in (**A**) fitted to simple linear regression model; comparisons in (**B**) were made using a linear model and in (**C**) from a linear mixed-effects model. ****P < 0.0001, ***P < 0.001, **P < 0.01, *P < 0.05, ns = not significant.

Next, we examined the relationship between IgG reactivity and the abundance of *P. falciparum* merozoite proteins. To do so, we used MS to determine the quantity of proteins expressed by merozoites purified from culture. On the microarray, 281 proteins/polypeptides represented 251 of the full-length merozoite proteins for which MS data was available. We observed a positive correlation between IgG reactivity and merozoite protein abundance (*P* < 0.001; Figure 5A). We then classified the same 281 proteins into high abundance (*N* = 68) and low abundance proteins (*N* = 213) by fitting a mixture of densities model and found that IgG reactivity was significantly greater against more highly abundant proteins (*P* < 0.01; Figure 5B)—a pattern that was consistently observed within each of the age strata (*P* < 0.001; Figure 5C).

### Protein size correlates inversely with IgG reactivity.

Next, we investigated the relationship between IgG reactivity and the MW of proteins on the microarray. Because the microarray contains both full-length proteins and polypeptides (in the case of larger proteins), we examined the relationship between IgG reactivity and the MW of each protein represented on the microarray. We observed an inverse correlation between IgG reactivity and MW (*P* < 0.001; Supplemental Figure 1A). We then classified proteins into high (*N* = 482) and low MW (*N* = 604) by fitting a mixture of densities model. We found that IgG reactivity to high MW proteins was significantly lower than IgG reactivity to low MW proteins (*P* < 0.01; Supplemental Figure 1B), a pattern that was again apparent across the age strata (*P* < 0.001; Supplemental Figure 1C).

### IgG reactivity to proteins with human orthologs is significantly lower.

**Figure 6.**
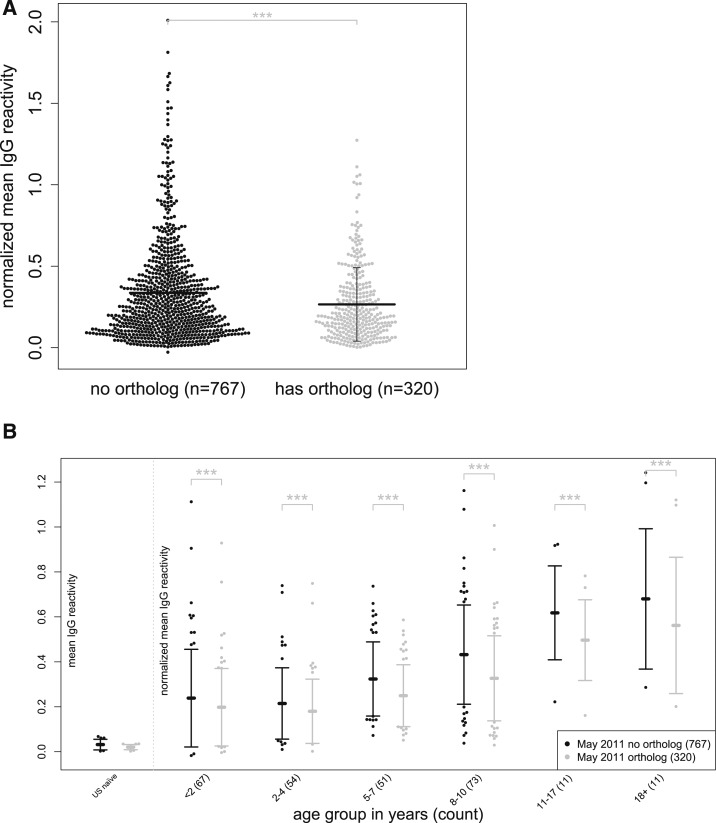
Immunoglobulin G (IgG) reactivity is lower to *Plasmodium falciparum* proteins with human orthologs. (**A**) Mean IgG reactivities to *P. falciparum* proteins with (*N* = 320) or without (*N* = 767) human orthologs. Vertical bars represent standard deviation from mean. (**B**) Mean IgG reactivities to *P. falciparum* proteins with (*N* = 320) or without (*N* = 767) human orthologs stratified by age. All data are normalized to mean IgG reactivity of U.S. adults. Comparisons in (**A**) were made using a linear model and in (**B**) a linear mixed-effects model. ****P < 0.0001, ***P < 0.001, **P < 0.01, *P < 0.05, ns = not significant.

Next, we tested the hypothesis that IgG responses are lower to *P. falciparum* proteins predicted to have human orthologs, consistent with the deletion of autoreactive B- and T-lymphocyte clones. Using the orthoMCL algorithm, which clusters proteins into ortholog groups by BLAST similarity,^26^ we identified 256 full-length *P. falciparum* proteins (represented by 320 microarray proteins) with predicted human orthologs. Consistent with our hypothesis, IgG reactivity to *P. falciparum* proteins with predicted human orthologs was significantly lower than IgG reactivity to those without predicted human orthologs (*P* < 0.001; Figure 6A), a finding also observed across the age strata (*P* < 0.001; Figure 6B).

### Degree of polymorphism correlates with IgG reactivity.

**Figure 7.**
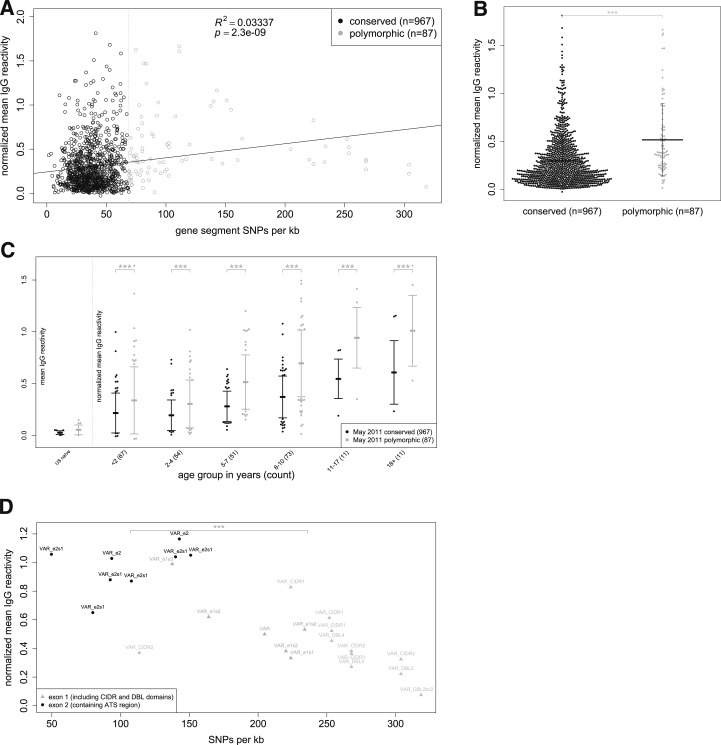
Immunoglobulin G (IgG) reactivity correlates with degree of protein polymorphism. (**A**) Scatterplot showing mean IgG reactivity (protein microarray data for 267 subjects) vs. single nucleotide polymorphism (SNPs)/kb for each of 1054 proteins/polypeptides (dots). (**B**) Mean IgG reactivities to conserved (*N* = 967) vs. polymorphic proteins (*N* = 87). Vertical bars represent standard deviation from mean. (**C**) Mean IgG reactivities to conserved (*N* = 967) vs. polymorphic proteins (*N* = 87) stratified by age. (**D**) Scatterplot showing mean IgG reactivity to *P. falciparum* erythrocyte membrane protein 1s (*Pf*EMP1s) on the microarray (protein microarray data for 267 subjects) vs. SNPs/kb for each of the *Pf*EMP1s (dots). All data are normalized to mean IgG reactivity of U.S. adults. Scatterplot in (**A**) fitted to simple linear regression model; comparisons of reactivity in (**B**) were made using a linear model, in (**C**) from a linear mixed-effects model, and in (**D**) with a Welch Two Sample *t* test. The SNPs/kb were calculated using both synonymous and nonsynonymous SNPs. ****P < 0.0001, ***P < 0.001, **P < 0.01, *P < 0.05, ns = not significant.

We examined the relationship between IgG reactivity and the degree of polymorphism of *P. falciparum* proteins. For each protein or polypeptide on the microarray we determined the number of single nucleotide polymorphism (SNPs)/kb in the corresponding gene or gene fragment, respectively.^18^ We observed a positive correlation between IgG reactivity and SNPs/kb (*P* < 0.001; Figure 7A). Next, we used a mixture of densities model to classify microarray proteins as conserved (*N* = 967) or polymorphic (*N* = 87) and found that average IgG reactivity is higher to polymorphic proteins (*P* < 0.001; Figure 7B), a pattern consistent across age groups (*P* < 0.001; Figure 7C). As expected, IgG reactivity only varied with the number of nonsynonymous SNPs/kb (*P* < 0.001; Supplemental Figure 2B), whereas no correlation was found between IgG reactivity and the number of synonymous SNPs/kb (*P* < 0.87; Supplemental Figure 2A).

We then focused on the antibody response to *P. falciparum* erythrocyte membrane protein 1 (*Pf*EMP1). Each parasite genome contains ∼60 *var* genes encoding the highly polymorphic PfEMP1s that are clonally expressed by the parasite and transported to the host erythrocyte plasma membrane.^29^ PfEMP1s bind to ligands on endothelial cells to sequester infected erythrocytes in the venules of various tissues to escape splenic clearance. Eight polypeptides (with a mean of 107.1 SNPs/kb 95% confidence interval [CI]: 77.6–136.6) on the microarray correspond to exon 2 of PfEMP1, which encodes the relatively conserved intraerythrocytic Acidic Terminal Segment (ATS), whereas 19 polypeptides (with a mean of 235.9 SNPs/kb 95% CI: 206.8–265.0) represent the highly polymorphic extraerythrocytic domains expressed from exon 1. In contrast to the correlation observed between IgG reactivity and SNPs/kb for all proteins on the array (Figure 7A), IgG reactivity was higher against the conserved ATS domains compared with the polymorphic extraerythrocytic domains (*P* < 0.001; Figure 7D).

### Polymorphism in plasma membrane proteins predicts IgG reactivity in multiple regression analysis.

Finally, we constructed multiple linear regression models to identify proteomic variables that are independently associated with IgG reactivity. Pearson χ^2^ testing for independence between factors demonstrated significant interactions between the following variables: polymorphism*subcellular location (*P* < 0.001), polymorphism*human orthologs (*P* < 0.001), subcellular location*human orthologs (*P* < 0.001), and MW*human orthologs (*P* < 0.05) (Table 2). Protein abundance as a variable was excluded from the model because only data from the merozoite stage of the parasite life cycle was available. Bidirectional stepwise regression indicated all four proteomic variables and three of the interaction terms (polymorphisms*subcellular location, polymorphisms*human orthologs, and MW*human orthologs) should be included in the model. Given that subcellular location had a limited number of exclusively assigned antigens (608 of 1,087, or 56%) and the implied limitation of significant interaction of subcellular location to just polymorphisms by stepwise regression, we constructed two models: the first considering only subcellular location and polymorphisms as independent variables and the second without subcellular location but with all other variables and their significant interactions. In the final model, with subcellular location and polymorphisms (*R*^2^ = 0.199), IgG reactivity significantly increased with additional SNPs/kb among proteins located in the plasma membrane (*P* < 0.0001) but no significant relationship was noted for other subcellular locations (Supplemental Figure 3). Of note, no plasma membrane proteins had SNPs/kb greater than 120. In the second model without subcellular location, we noted a low *R*^2^ of 0.0685 suggesting a minimal role of degree of polymorphism, presence of human ortholog, or MW as determinants of IgG reactivity. We validated this model with protein microarray data generated from the same cohort (*N* = 229 subjects) using plasma samples collected 1 year later in May 2012, and again noted a significant increase in IgG reactivity with additional SNPs/kb among plasma membrane proteins as well as similar *R*^2^ values (*R*^2^ = 0.172 in first model, 0.0572 in second model, Table 3).

**Table 2:** Pearson’s χ^2^ testing for independence

	Subcellular location	Presence of human ortholog	High molecular weight
High degree of polymorphism	χ^2^: 124.6626****	χ^2^: 19.7439****	χ^2^: 1.7487
	df: **2**	df: **1**	df: 1
	*P* value: **< 2.2e−16**	*P* value: **8.854e−06**	*P* value: 0.186
			
Subcellular location	–	χ^2^: 29.7157****	χ^2^: 0.26634
		df: **2**	df: 2
		*P* value: **3.526e−07**	*P* value: 0.8753
			
Presence of human ortholog	–	–	χ^2^: 4.0901*
			df: **1**
			*P* value: **0.04313**

****P < 0.0001, ***P < 0.001, **P < 0.01, *P < 0.05.

**Table 3:** Determinants of IgG reactivity in May 2011 and 2012 by multiple regression analysis

Coefficient	May 2011		May 2012	
	Estimate	Pr (> |*t*|)	Estimate	Pr (> |*t*|)
**(Intercept)**	5.44E−01****	**< 2.00E−16**	4.53E−01****	**< 2.00E−16**
Segment SNPs per kb	−4.07E**−**05	9.12E**−**01	−2.15E**−**04	4.97E**−**01
**Location [intracellular region]**	−3.37E−01****	**3.30E−10**	−2.81E−01****	**7.57E−10**
**Location [plasma membrane]**	−7.70E−01****	**7.47E−12**	−6.17E−01****	**1.08E−10**
Segment SNPs per kb*location [intracellular region]	1.46E**−**03	1.01E**−**01	1.39E**−**03	6.75E**−**02
Segment SNPs per kb*location [plasma membrane]	1.43E−02****	**3.18E−11**	1.13E−02****	**7.75E−10**
Multiple *R*^2^	0.199		0.172	
**(Intercept)**	2.99E−01****	**< 2e−16**	2.54E−01****	**< 2e−16**
**Segment SNPs per kb**	1.75E−03****	**4.07E−10**	1.28E−03****	**7.10E−08**
Has ortholog	−3.13E**−**02	4.37E**−**01	−3.02E**−**02	3.74E**−**01
**MW**	−2.67E−07****	**8.58E−08**	−2.23E−07****	**1.32E−07**
Has ortholog*MW	3.00E−07***	**3.95E−04**	2.56E−07***	**3.56E−04**
Segment SNPs per kb*has ortholog	−1.99E−03*	**4.10E−02**	−1.46E**−**03	7.64E**−**02
Multiple *R*^2^	0.0685		0.0572	

IgG = immunoglobulin G; MW = molecular weight; SNP = single nucleotide polymorphism. Estimations of coefficients in two multiple regression models of normalized mean IgG reactivity with covariates of SNPs/kb and subcellular location in the first model, and presence of ortholog, molecular weight, and SNPs/kb in linear combination with interaction variables identified by Pearson χ^2^ analysis and bidirectional stepwise regression from 267 subjects in May 2011 and 234 of the same subjects in May 2012.

****P < 0.0001, ***P < 0.001, **P < 0.01, *P < 0.05.

## DISCUSSION

Here, we sought to gain insight into protein-specific factors that underlie heterogeneity in the magnitude of IgG responses to *P. falciparum* antigens in the context of natural infection—a study made possible by genomics-based technology that allows antibody responses to be interrogated across the entire or large portions of the proteome of important pathogens. When not accounting for interactions, we observed that IgG responses were higher to extracellular and plasma membrane proteins and correlated positively with both protein abundance and degree of protein polymorphism. By contrast, IgG reactivity was significantly lower to proteins predicted to have human orthologs and correlated inversely with protein MW.

The observation that IgG responses are higher to extracellular and plasma membrane *P. falciparum* proteins is consistent with recent studies using protein microarrays to profile IgG responses in individuals with tuberculosis^11^ or brucellosis.^12^ The link between the magnitude of antibody responses and protein location is also consistent with experimental data in mouse models. For example, surface display of *Plasmodium* antigen on *Salmonella typhimurium* induced higher antibody responses than periplasmic expression of the same antigen.^30^ Similarly, B cells demonstrated a greater ability to respond to help from influenza-specific T-cells when viral antigens were external versus internal.^31^

The finding that IgG responses to *P. falciparum* merozoite proteins correlated with the abundance of the same merozoite proteins in vitro is consistent with the same brucellosis study, which showed that immunodominant antigens are enriched with proteins that are expressed at higher levels in vitro.^12^ However, this finding should be interpreted with caution as our analysis was restricted to merozoites. Moreover, merozoite protein abundance in vitro may not accurately reflect in vivo abundance.

We observed that IgG reactivity was higher to polymorphic proteins (greater number of nonsynonymous SNPs/kb). However, multivariate regression analysis suggests that the relationship between IgG reactivity and protein polymorphism is confounded by the finding that plasma membrane and extracellular proteins tend to be more polymorphic. In other words, higher IgG responses to extracellular and plasma membrane proteins may be driven by accessibility (as suggested by the murine studies noted previously), and presumably this same antibody response drives extracellular/plasma membrane proteins to become polymorphic by impacting parasite fitness.

This point is highlighted by contrasting IgG responses to the intra- and extraerythrocyte domains of the *var* gene-encoded PfEMP1s. Exon 2 of the *var* genes encodes the relatively conserved intraerythrocyte ATS domain, which anchors *Pf*EMP1 in the erythrocyte membrane,^32^ whereas exon 1 encodes the hypervariable domains duffy binding-like domain (DBL) and cysteine-rich interdomain region (CIDR), which are expressed on the erythrocyte surface and mediate sequestration of infected erythrocytes in the host vasculature to avoid splenic clearance. Although more conserved, IgG reactivity was greater to the intraerythrocyte ATS domain compared with the extraerythrocyte DBL and CIDR domains of the same protein. That the intraerythrocyte ATS domain is conserved is consistent with it being accessible to host antibody responses only after the parasite has completed its 48-hour cycle of erythrocyte invasion, replication and rupture. This example highlights the requirement that antibody responses decrease parasite fitness for high degrees of polymorphism to arise from natural selection and also underscores the lack of causality between the immunogenicity of a given protein and its degree of polymorphism.

We found that IgG reactivity to *P. falciparum* proteins with predicted human orthologs was significantly lower than IgG reactivity to proteins without predicted human orthologs, consistent with the deletion of autoreactive B- and T-lymphocyte clones in the host. An interesting corollary to this finding is the possibility that the host species can lose the ability to express self-antigens through selective pressure if it results in a gained ability to mount protective antibody responses against similar antigens expressed by pathogens.^33^

In this analysis, we found that MW correlated inversely with IgG reactivity. However, this finding should be interpreted with caution as larger proteins may be expressed less efficiently in the *E. coli*-based cell-free expression system used to construct the protein microarrays.^34^

In summary, we found that IgG reactivity was significantly higher to extracellular and plasma membrane proteins and also correlated positively with both protein abundance and degree of protein polymorphism, although the relationship between IgG reactivity and polymorphisms may be confounded by protein subcellular location. Conversely, IgG reactivity was significantly lower to proteins predicted to have human orthologs. These findings provide insight into protein-specific factors that are associated with variability in the magnitude of antibody responses to natural *P. falciparum* infection—data that could inform vaccine strategies to optimize antibody-mediated immunity as well as the selection of antigens for sero-diagnostic purposes.

## Supplementary Material

Supplemental Figure.
