# Erratum

**DOI:** 10.4269/ajtmh.17-0437err

**Published:** 2018-02

In *Am J Trop Med Hyg 98(1):* 57–66 by Lui et al (https://doi.org/10.4269/ajtmh.17-0437) there is an error in the text. In the first sentence of the “Experimental design and statistical rationale” heading on page 58 it reads “A total of 515 experimental plasma samples (267 from May 2011, 234 from May 2012, and 14 replicates) were probed by protein microarray.” It should instead read “A total of 508 experimental plasma samples (267 from May 2011, 229 from May 2012, and 12 replicates) were probed by protein microarray.”

