# Supplementary material for: Protein-Specific Features Associated with Variability in Human Antibody Responses to Plasmodium falciparum Malaria Antigens
Source: Am J Trop Med Hyg. 2017 Oct 23;98(1):57–66. doi: 10.4269/ajtmh.17-0437 (PMC5928716; doi:10.4269/ajtmh.17-0437)
Supplement: Supplementary file 1 [file tpmd170437.SD1.pdf]

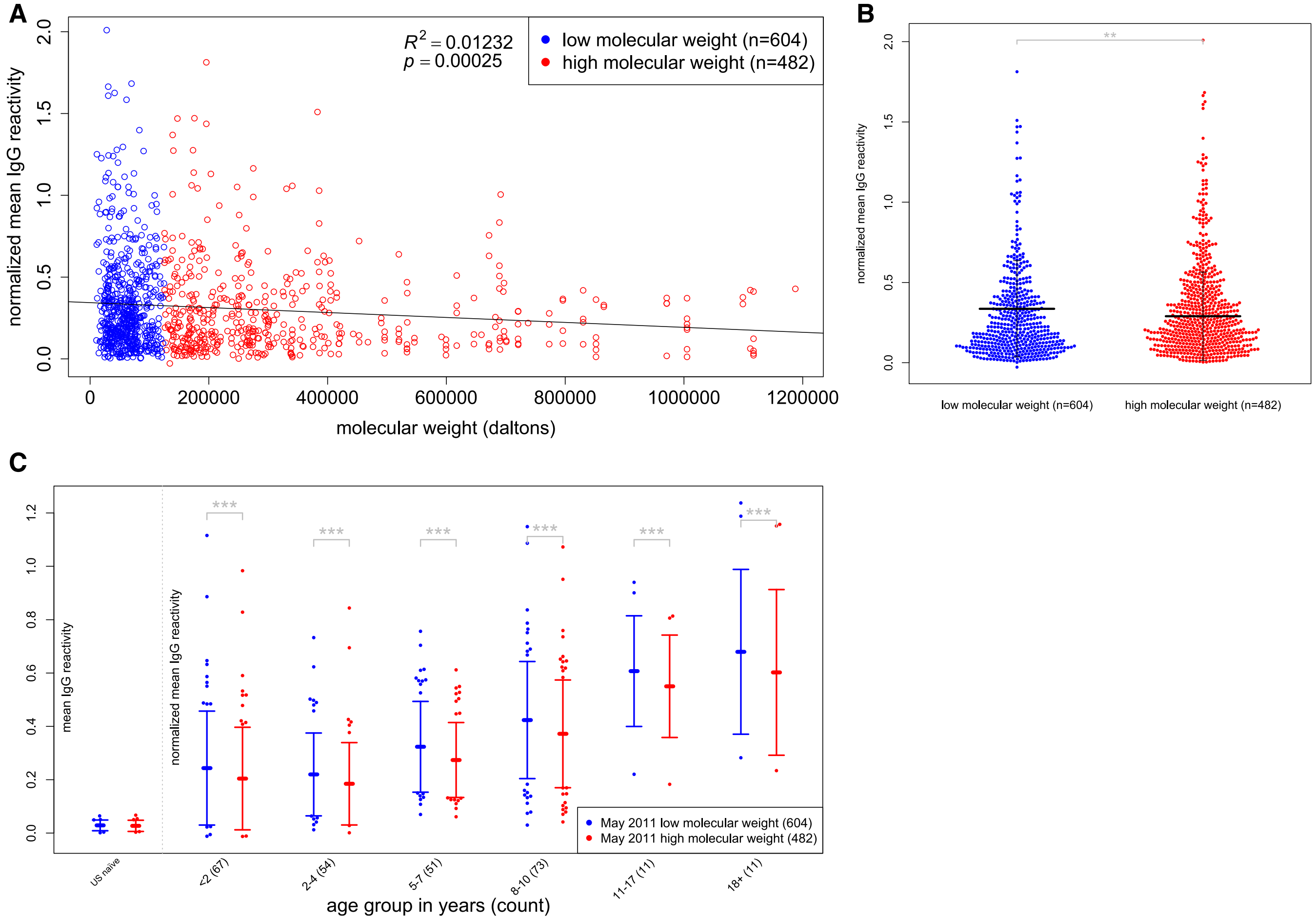

Supplemental Figure 1. Molecular weight of associated full-length protein is inversely correlated with reactivity. (**A**) Scatterplot comparison of mean Immunoglobulin G (IgG) reactivities normalized to mean IgG reactivity of US naives for each microarray protein by molecular weight of associated full-length protein. Blue points represent proteins with low molecular weight and red points those with high molecular weight by a mixture of densities model. (**B**) Comparison of normalized mean IgG reactivities by low (blue) or high (red) molecular weight. Vertical bars represent standard deviation from mean of reactivities (bold horizontal line). (**C**) Mean plot with outliers one standard deviation from the mean of normalized mean IgG reactivities for proteins with low (blue) or high (red) molecular weight stratified by age group. Comparisons were made from a linear model in (**B**) and from a linear mixed-effects statistical model in (**C**). ****$P < 0.0001$, ***$P < 0.001$, **$P < 0.01$, *$P < 0.05$, ns = not significant.

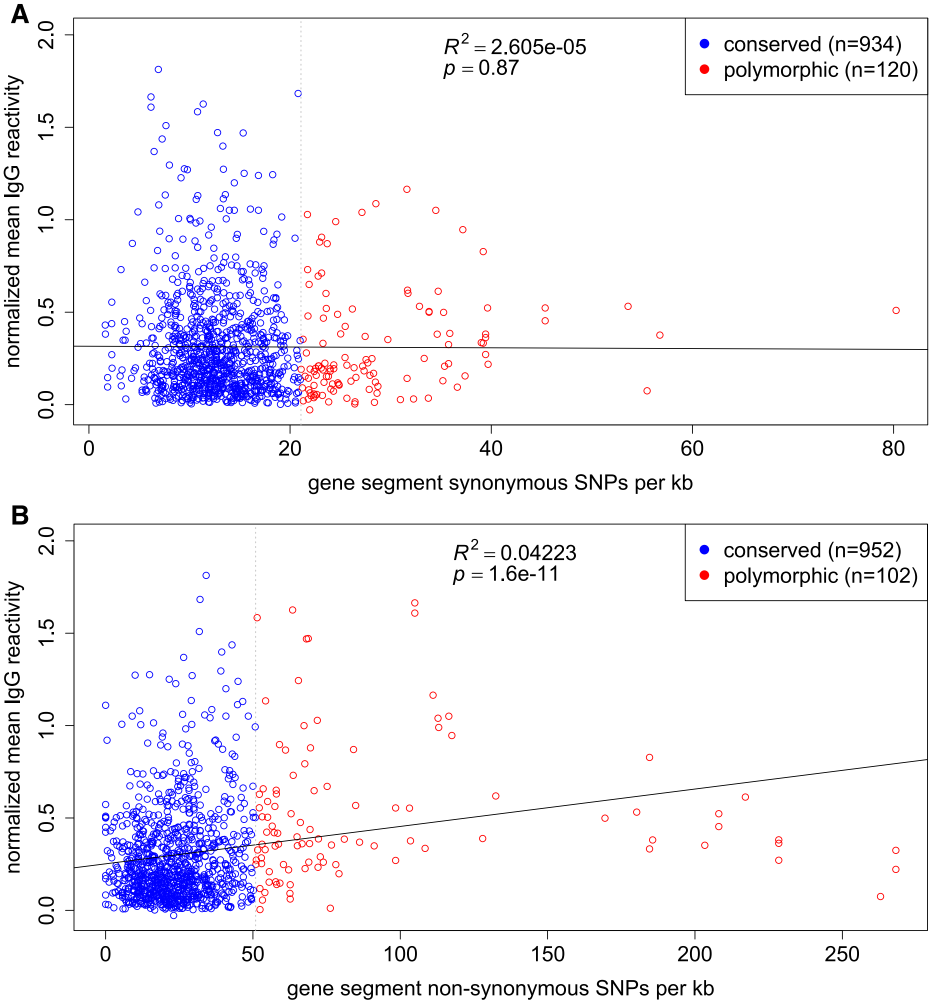

Supplemental Figure 2. Reactivity is not correlated with degree of polymorphism when measured with synonymous single nucleotide polymorphism (SNPs)/kb, but is correlated when measured with nonsynonymous SNPs/kb. (**A**) Scatterplot comparison of mean immunoglobulin G (IgG) reactivities normalized to mean IgG reactivity of US naives by number of synonymous SNPs/kb of 1,054 proteins in 267 subjects May 2011. (**B**) Scatterplot comparison of mean IgG reactivities normalized to mean IgG reactivity of US naives by number of nonsynonymous SNPs/kb in the same subjects May 2011.

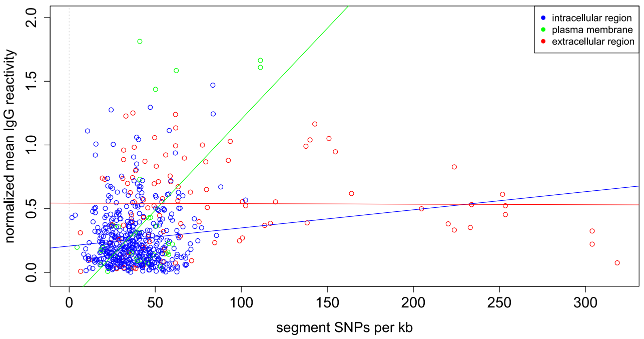

Supplemental Figure 3. On multiple regression analysis, correlation between degree of polymorphism and Immunoglobulin G (IgG) reactivity is dependent on subcellular location. Scatterplot comparison of mean IgG reactivities normalized to mean IgG reactivity of US naives by number of single nucleotide polymorphism (SNPs)/kb of 589 proteins in 267 subjects May 2011 with stratification by protein subcellular location. Proteins mapped exclusively to the intracellular region are shown in blue, the plasma membrane in green, and extracellular region in red.
